# Treatment Strategies for Advanced Cutaneous Squamous Cell Carcinoma Following Anti-PD-1 Immunotherapy Failure: A Systematic Review of the Literature and Ongoing Trials

**DOI:** 10.3390/cancers18183060

**Published:** 2026-09-21

**Authors:** Hamish Thomson, Embiye Adala, Anirban Mandal, Christopher Jones, Joseph J. Sacco

**Affiliations:** 1 Ninewells Hospital, NHS Tayside, Dundee DD2 1UB, UK; hamish.thomson@nhs.scot; 2 The Mersey Regional Burns and Plastic Surgery Unit, Mersey and West Lancashire Teaching Hospitals NHS Trust, Prescot L35 5DR, UK; embiye.mba@gmail.com (E.A.); anirban.mandal@merseywestlancs.nhs.uk (A.M.); christopher.jones@merseywestlancs.nhs.uk (C.J.); 3 The Clatterbridge Cancer Centre, Liverpool L7 8YA, UK; 4 School of Medicine, University of Liverpool, Liverpool L69 3BX, UK

**Keywords:** cutaneous squamous cell carcinoma, unresponsive to anti-PD-1 immunotherapy, advanced, management

## Abstract

**Background:** While anti-PD-1 immunotherapy has led to significant improvements in the outcome of advanced cutaneous squamous cell carcinoma (cSCC), over half of patients fail to respond, and there remains no established second-line therapy, representing a clear unmet need. **Aim/Methods:** Here, we present a systematic review of the literature and ongoing clinical trials to evaluate current evidence on treatment strategies in this setting. Following a comprehensive search, and quality control assessment of each paper, we identified 20 studies that were included in the final review: 11 published papers, 1 conference poster and 8 ongoing clinical trials. Data from the 12 reported studies were presented in a study-by-study format. Outcomes were reported descriptively and included the overall response rate (ORR), complete response (CR), partial response (PR), stable disease (SD) and progressive disease (PD), progression-free survival (PFS) and, where available, overall survival (OS). **Results:** Although the review is limited by small sample sizes and the frequent use of combination rather than monotherapy regimens, the evidence suggests that epidermal growth factor receptor (EGFR) inhibitor-containing treatment strategies are the most frequently reported salvage approach and demonstrate preliminary evidence of treatment activity in advanced cSCC following anti-PD-1 failure. **Conclusions:** However, current evidence is insufficient to establish superiority over alternative treatments. Other treatment strategies, such as ipilimumab, oncolytic virotherapy and chemo-immunotherapy, show clinical promise warranting further investigation.

## Simple Summary

Cutaneous squamous cell carcinoma is a common type of skin cancer that is usually cured with surgery. However, some patients develop advanced disease that cannot be removed surgically or spreads to other parts of the body. Although immunotherapy has improved outcomes, many patients do not respond to treatment or their cancer progresses, and there is currently no clear evidence to guide the next stage of treatment. This systematic review brings together the available research on treatments used after immunotherapy failure and examines ongoing clinical trials investigating new approaches. The findings suggest that epidermal growth factor receptor-containing strategies are the most frequently reported salvage approach and demonstrate preliminary evidence of treatment activity. However, the available evidence is limited by small, heterogeneous, non-randomised cohorts and is insufficient to establish superiority over alternative treatments. Several other promising immunotherapy-based strategies are also under investigation. This review provides an up-to-date summary of the available evidence to support clinical decision-making and help guide future research in this challenging area.

## 1. Introduction

Cutaneous squamous cell carcinoma (cSCC) is the second most common skin malignancy and represents an increasing global cancer burden, with a rising incidence, particularly in ageing populations and high-sociodemographic-index regions. The 2021 Global Burden of Disease database recorded over 1.8 million cases across 204 countries [1]. In the UK, approximately 25,000 new cases are diagnosed annually [2,3] with the increasing disease burden attributable to factors including ultraviolet (UV) radiation exposure, an ageing population and the increasing use of long-term immunosuppressive medications [4]. Although approximately 95% of cSCC cases are cured with local treatment, including ablation, wide local excision, or Mohs surgery, a small proportion of patients develop unresectable, locally advanced, or metastatic disease [5,6]. These patients are candidates for systemic immunotherapy, for which the current first-line treatment is immune checkpoint inhibition, specifically anti-programmed cell death protein 1 (anti-PD-1) inhibitors such as pembrolizumab, cemiplimab, and nivolumab [6].

Anti-PD-1 inhibitors are monoclonal antibodies that restore anti-tumour immunity by blocking the interaction between PD-1 on cytotoxic CD8+ T cells and programmed death-ligand 1 (PD-L1) expressed within the tumour microenvironment. This inhibits tumour-induced T-cell exhaustion and enhances cytotoxic immune-mediated tumour cell killing [7]. In the recent phase II EMPOWER-CSCC-1 trial, cemiplimab demonstrated overall response rates (ORRs) of 47.2% with weight-based dosing and 44.8% with fixed dosing, with a median time to complete response of 4.2 months in patients with locally advanced or metastatic cSCC [8]. However, a remaining ~50% of patients are primarily resistant to anti-PD-1 therapy or develop disease progression during or following treatment [5]. Proposed mechanisms of resistance include the presence of an immunosuppressive tumour microenvironment enriched with regulatory T-cells (Tregs), myeloid-derived suppressor cells (MDSCs), tumour-associated macrophages (TAMs), and cancer-associated fibroblasts (CAFs). Such microenvironments may be particularly relevant in patient groups exposed to long-term immunosuppression, such as solid organ transplant recipients [9]. Perineural invasion has also been identified as a possible predictor of anti-PD-1 resistance [10].

Second-line management options for advanced cSCC following anti-PD-1 failure remain unclear, with no large studies to guide decisions. With 40–50% of patients requiring second-line therapy, an evidence-based synthesis of the available management strategies is needed to support oncological decision-making in anti-PD-1-refractory cSCC. Reported approaches include further immunotherapy, chemotherapy, epidermal growth factor receptor (EGFR) inhibition, cytotoxic T-lymphocyte-associated protein-4 (CTLA-4) inhibition, toll-like receptor (TLR9) agonism, and human epidermal growth factor receptor 2 (HER2)-directed therapy [11,12,13,14,15]. EGFR inhibitors, such as cetuximab, block the activation of EGFR, which is frequently overexpressed in cSCC, thereby inhibiting downstream signalling pathways involved in tumour proliferation, including the mitogen-activated protein kinase (MAPK) and phosphatidylinositol 3-kinase/protein kinase B (PI3K/AKT) pathways. Through suppression of EGFR-mediated signalling, these agents may reduce tumour growth in advanced cSCC [11]. CTLA-4 inhibitors, such as ipilimumab, block the inhibitory CTLA-4 receptor on T cells, thereby enhancing T-cell activation and promoting immune-mediated tumour cell destruction [12,13]. Toll-like receptor 9 (TLR9) agonists, such as cavrotolimod, stimulate innate immune responses through plasmacytoid dendritic cells and B cells, inducing interferon signalling, granzyme release, and natural killer cell-mediated cytotoxicity. This may enhance T-cell activation while suppressing immunosuppressive cell populations, thereby promoting anti-tumour immunity [14]. HER2-directed therapies have also been reported, although evidence remains limited. HER2 signalling in cSCC appears to occur primarily through HER3-mediated activation of the MAPK and PI3K/AKT pathways. Agents such as lapatinib inhibit this tyrosine kinase signalling and may therefore have therapeutic potential in selected cases of advanced cSCC [15]. Oncolytic virotherapies are genetically engineered viruses designed to selectively infect and destroy cancer cells whilst concurrently stimulating a systemic anti-tumour response. For example, vusolimogene oderparepvec (RP1) is based on the herpes simplex virus 1 (HSV-1) and selectively replicates within cancer cells causing immunogenic cell death, whilst driving expression of granulocyte-macrophage colony-stimulating factor (GM-CSF) to stimulate a systemic anti-tumour response [16].

Less frequently reported salvage approaches include chemo-immunotherapy and non-conventional PD-1 blockade. Rahimi et al. described the addition of carboplatin and paclitaxel to continued anti-PD-1 therapy following primary resistance, providing a potential rationale for combining cytotoxic chemotherapy with immune-checkpoint inhibition in an attempt to overcome treatment resistance [17]. Dostarlimab, another monoclonal antibody targeting PD-1, has also been reported following cemiplimab failure in an individual case of advanced cSCC [18].

This systematic review adhered to the PRISMA guidelines [19] and followed the population, intervention, comparison, outcomes and time (PICOT) framework [20]. The review aimed to evaluate the current medical literature on treatment strategies for advanced cSCC in patients who were unsuitable for anti-PD-1 immunotherapy or who developed resistance, treatment failure, or disease progression following its initiation. The review was undertaken to provide an evidence-based reference for multidisciplinary teams involved in the management of patients with advanced cSCC following anti-PD-1 immunotherapy failure.

## 2. Materials and Methods

This review was registered on PROSPERO [21] on 23 March 2026 (ID: CRD420261340230) [22]. A systematic search of the medical databases PubMed, MEDLINE and Scopus was conducted on 27 August 2026, adhering to the PRISMA guidelines [19] and using separate search strategies adapted to each database. The searches were limited to studies published between 1 January 2000 and 27 August 2026 to ensure relevance to contemporary immunotherapy and modern management of cSCC. Four groups of keywords and MeSH terms were used to synthesise the search strategies: (1) advanced cutaneous squamous cell carcinoma; (2) anti-PD-1 immunotherapy; (3) treatment resistance; and (4) salvage treatment strategies. The specific search terms and Boolean operators used for each database are specified in Tables S1–S3 within Supplementary Materials. Ongoing trials were identified by searching ClinicalTrials.gov [23] on 27 August 2026 using the search term “cutaneous squamous cell carcinoma” and filtering the recruitment status to “Recruiting”, “Not yet recruiting”, and “Active, not recruiting”.

The selection criteria were guided by the PICOT framework [20] as outlined in the introduction, to support a consistent comparison of treatment modalities and outcomes following anti-PD-1 immunotherapy failure in advanced cSCC:

- Population (P)—Patients undergoing treatment for advanced cSCC who were unsuitable for, suffered disease progression on, or were previously resistant to anti-PD-1 immunotherapy.
- Intervention (I)—Any systemic anti-cancer therapy subsequently used in the event of resistance or disease progression following treatment of advanced cSCC with anti-PD-1 immunotherapy.
- Comparison (C)—Surgery, intra-tumoral therapy, photoimmunotherapy, radiotherapy or any other systemic anti-cancer therapy subsequently used in the event of resistance or disease progression following treatment of advanced cSCC with anti-PD-1 immunotherapy.
- Outcomes (O)—(a) Anti-cancer agent utilised following anti-PD-1 failure, (b) overall survival (OS) (c) progression-free survival (PFS), and (d) response rate (Complete Response (CR)/Partial Response (PR), Stable Disease (SD) or Progressive Disease (PD)).
- Time (T)—Both the short term (≤1 year) and long term (≥1 year).

Following the search, all identified studies were uploaded to the latest version (as of January 2026) of the AI systematic review software Rayyan [24], and duplicate papers were removed, after which two authors independently assessed the titles and abstracts of each study against the inclusion and exclusion criteria. Additionally, the bibliography list of each article was screened to identify any further potentially relevant studies. Studies were restricted to reports published in English. Each study was required to have an identifiable study design, clearly indicate that anti-PD-1 immunotherapy had been unsuccessful in treating patients’ advanced cSCC, and show what subsequent treatment was used following anti-PD-1 failure alongside relevant outcomes. Additionally, systematic reviews and papers published prior to 1 January 2000 and/or outside the scope of the review were excluded.

**Figure 1:**
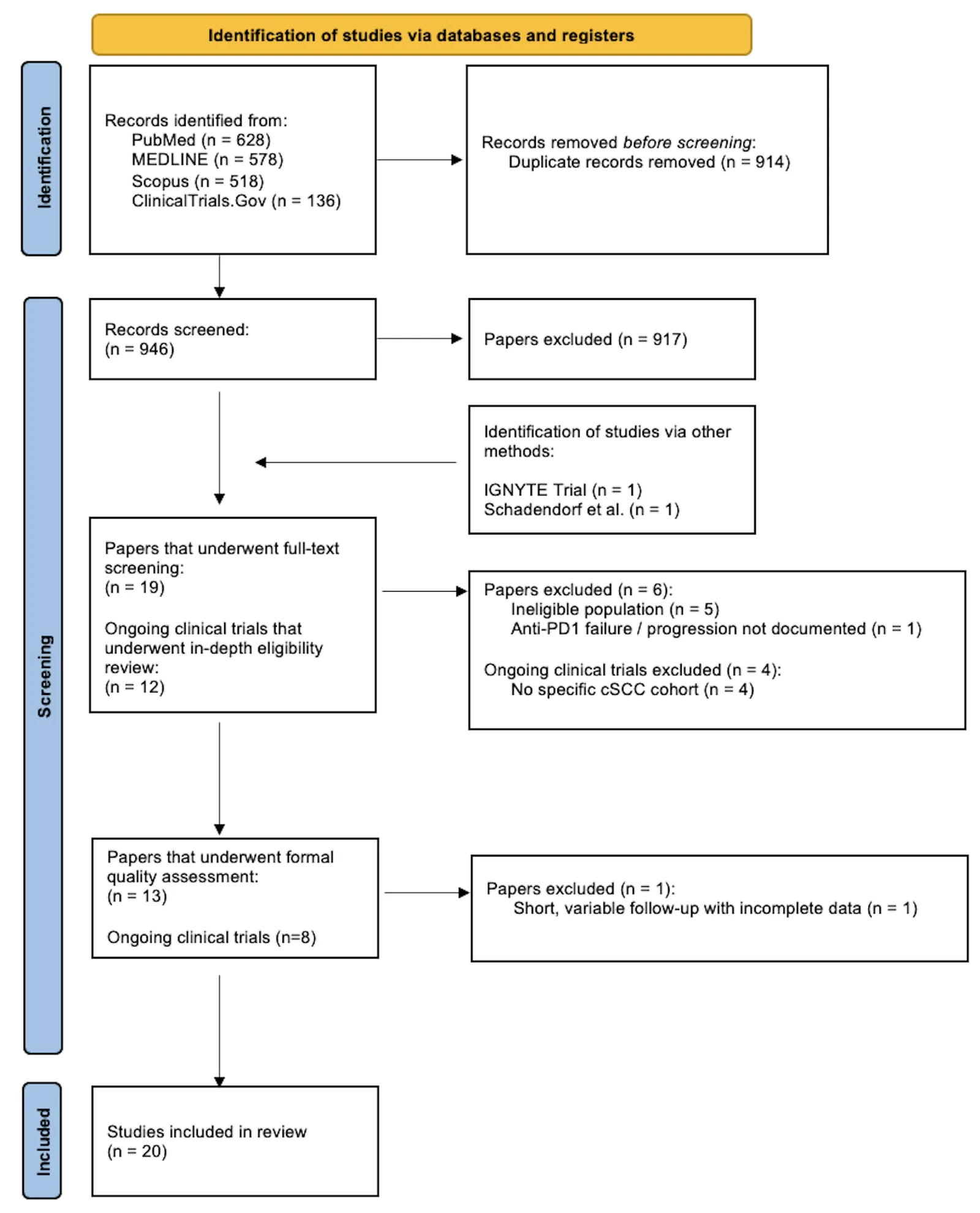
PRISMA flow diagram illustrating record identification, screening, eligibility assessment, quality assessment and final study inclusion.

As outlined in Figure 1, the initial search identified 1860 records (PubMed = 628, MEDLINE = 578, Scopus = 518, ClinicalTrials.gov = 136). Following the removal of 914 duplicates, 946 papers remained for independent screening by two authors. Of these, 917 were excluded for failing to meet the inclusion criteria. During the screening process, a further relevant ongoing trial was identified through screening bibliographies, although this trial was not identified by the original search of ClinicalTrials.gov due to the search strategy not screening for the condition “Non-melanoma Skin Cancer” [25]. Additionally, a relevant conference poster from the 2026 European Association of Dermato-Oncology (EADO) conference was identified through author knowledge and included [26]. Following title and abstract screening, 19 completed reports (18 full-text publications and one conference poster) underwent full-text screening [4,5,6,14,15,17,18,26,27,28,29,30,31,32,33,34,35,36,37]. In parallel, 12 ongoing trial registrations underwent detailed eligibility assessment [25,38,39,40,41,42,43,44,45,46,47,48].

### 2.1. Full-Text Screening

All 19 papers in which a full text was available underwent full-text screening [4,5,6,14,15,17,18,26,27,28,29,30,31,32,33,34,35,36,37], during which 6 were found to be ineligible for inclusion [27,28,29,30,31,32]. Five studies were found to report on head and neck squamous cell carcinoma (HNSCC) more broadly rather than specifically on cSCC and were therefore excluded prior to quality assessment due to reporting on an ineligible population [27,28,30,31,32]. Kassardijan et al. was excluded as it was not explicitly stated whether patients had progressed on anti-PD-1 immunotherapy [29]. Ongoing clinical trials were not subjected to formal risk-of-bias assessment because outcome data were not yet available. Instead, each trial was independently reviewed to confirm eligibility, including evaluating the study population and design, the requirement for prior treatment failure or refractoriness, either specifically to anti-PD-1/PD-L1 therapy or to available standard-of-care systemic therapy, which for advanced cSCC, is currently centred on anti-PD-1 immunotherapy. Four trials were excluded for being based on a broader HNSCC or solid-tumour population without having a dedicated cSCC cohort; whilst cSCC was a permitted population, they did not represent a dedicated post-anti-PD-1-failure cSCC trial [38,41,45,46]. This left 8 ongoing clinical trials that were included in the final review [25,39,40,42,43,44,47,48].

### 2.2. Quality Assessment

Thirteen studies—comprising four prospective non-randomised single-arm interventional studies, one of which was presented on a conference poster [6,14,26,34], three retrospective analyses [4,5,33], and six case reports [15,17,18,35,36,37]—underwent formal quality assessment independently by two authors using appraisal tools appropriate for their respective study designs. Prospective non-randomised single-arm interventional studies were assessed using the Joanna Briggs Institute (JBI) Critical Appraisal Checklist for Quasi-Experimental Studies tool [49], retrospective analyses with the JBI Critical Appraisal Checklist for Cohort Studies [50], and case reports with the JBI Critical Appraisal Checklist for Case Reports [51]. Individual domains were assessed as ‘Yes’, ‘No’, ‘Unclear’ or ‘Not applicable’. No numerical summary score or fixed threshold was applied, as the number and nature of domains differ between JBI appraisal tools. Overall appraisal was based on the pattern and clinical importance of methodological concerns identified across the domains, with disagreements resolved by consensus between reviewers. Studies were excluded where methodological limitations were considered substantial enough to compromise interpretation of the cSCC-specific outcome data.

Reports comprising small numbers of individually described patients were assessed using the JBI Critical Appraisal Checklist for Case Reports [51] where the original publication was presented as a case report and did not constitute a systematically sampled or consecutively recruited cohort. This applied to Morecroft et al. (*n* = 3) and Rahimi et al. (*n* = 2), both of which presented individual patient-level clinical narratives and were published as case reports [17,35]. Huynh et al. was the only study excluded following overall critical appraisal, primarily because the relevant cSCC subgroup had very short and variable follow-up, incomplete follow-up was inadequately addressed, and standardised response-assessment criteria were not reported [4]. Traffic-light summary tables presenting the quality assessments for all studies are provided in Tables S4–S6 within the Supplementary Materials. Thirteen studies underwent quality assessment using the corresponding tool to the study design, after which 12 studies and 8 ongoing clinical trials were included in the final review [5,6,14,15,17,18,25,26,33,34,35,36,37,39,40,42,43,44,47,48].

## 3. Results

The characteristics of each study, including the country, sample size, study design, oncological intervention following anti-PD-1 failure and median follow-up duration alongside response criteria, timing of assessment and evaluable denominator, are summarised in Table S7. Patients that underwent subsequent treatment of advanced cSCC following anti-PD-1 failure were identified. Patient data, confounding factors, pathological factors, and clinical and oncological outcomes from each study were extracted and compiled into Version 16.108.2 of Microsoft Excel [52] in a study-by-study format. The summary of this data is presented in Table S8. The characteristics of each ongoing trial, including registration number, cSCC-specific cohort, requirement for prior anti-PD-1 failure, recruitment status, enrolment status (actual or estimated), and treatment strategy, are summarised in Table S9. Tables S7–S9 are listed in the Supplementary Materials.

### 3.1. Study Characteristics

Data were extracted and presented descriptively from twelve papers, including four prospective non-randomised single-arm interventional studies [6,14,26,34], two retrospective analyses [5,33], and six case reports [15,17,18,35,36,37]. Overall, 4 papers involved multiple centres [6,14,26,34]. Only patients who suffered disease progression whilst on anti-PD-1 immunotherapy for treatment of advanced cSCC were included in the sample sizes. Across the review, the median sample size was 4 participants (range 1–39), with a male predominance. Of the studies that adequately reported age, the median age was 72.5 years (range, 36–93), and the median follow-up was 18 months (range, 0.5–72). Confounding factors, primary site of cSCC, American Joint Committee on Cancer (AJCC) stage, prior anti-cancer therapy and best response, were extracted and reported descriptively. Response-assessment methodology was also extracted, including the criteria used, timing of assessment, and evaluable denominator where reported; substantial variation was observed between studies, with prospective studies generally using RECIST-based criteria and several case reports relying on clinical, radiological and/or pathological assessment. Eight ongoing clinical trials were also identified including one Phase I [40], four Phase II [25,43,44,47] and two Phase I/II [42,48] trials, alongside one prospective interventional study [39]. Seven of the ongoing trials were multi-centre [25,39,40,42,44,47,48].

### 3.2. Anti-Cancer Agent(s) Utilised Following Anti-PD-1 Failure

Seven classes of anti-cancer therapy with sufficient data were identified in the treatment of advanced cSCC following anti-PD-1 failure; EGFR inhibitors, CTLA-4 inhibitors, RP1 oncolytic virotherapy, TLR9 agonists, chemo-immunotherapy, alternative anti-PD-1 immunotherapy and HER2 antagonists. Seven studies comprising 66 patients used intravenous (IV) EGFR inhibitor therapy following anti-PD-1 failure [5,6,33,34,35,36,37]. Cetuximab was used in all but three patients, who received panitumumab [36]. Intravenous cetuximab was administered as monotherapy in 14 patients [5,33]. Thirty-nine patients received an EGFR inhibitor in combination with concurrent anti-PD-1 immunotherapy: cetuximab with pembrolizumab (*n* = 25), cetuximab with avelumab (*n* = 10), cetuximab with carboplatin and pembrolizumab (*n* = 1), panitumumab with pembrolizumab (*n* = 2), and panitumumab with nivolumab (*n* = 1) [6,34,35,36,37]. The remaining 13 patients received cetuximab in combination with carboplatin and paclitaxel (*n* = 6), radiotherapy (*n* = 6), or carboplatin alone (*n* = 1) [5,33,35].

The remaining treatment categories were each represented by a single study. Al-Sharif et al. was the only study to report the use of IV ipilimumab (CTLA-4 inhibitor), with 11 patients receiving monotherapy and 1 receiving concurrent anti-PD-1 therapy (nivolumab (*n* = 1)) [5]. Schadendorf et al. reported 39 patients treated with intra-tumoral (IT) oncolytic virotherapy (RP1 (*n* = 39)) in combination with anti-PD-1 therapy (nivolumab (*n* = 39)) [26]. Milhem et al. described five patients who were treated with IT cavrotolimod (TLR9 agonist), all of whom received concurrent anti-PD-1 immunotherapy (cemiplimab (*n* = 5)) [14]. Rahimi et al. reported two patients treated with IV carboplatin and paclitaxel in combination with anti-PD-1 immunotherapy (pembrolizumab (*n* = 1), cemiplimab (*n* = 1)) [17]. Gandarillas et al. described one patient treated with IV dostarlimab monotherapy as an alternative anti-PD-1 agent [18]. Finally, Strickley et al. reported one patient treated with oral lapatinib (HER2 antagonist) in combination with anti-PD-1 therapy (nivolumab (*n* = 1)) [15].

### 3.3. Overall Survival

Of the seven studies reporting EGFR-containing therapy, Al-Sharif et al. and Becker et al. were the only studies to report OS, with median values of 41.7 weeks (range, 12.2–218.6+) and 120 weeks (95% CI, 44.8-NR), respectively [5,34]. The upper limit of the 95% confidence interval was not reached in Becker et al., while OS was not reported in the remaining studies examining EGFR-containing therapy [6,33,34,35,36,37]. Al-Sharif et al. also reported a median OS of 45 weeks (range, 7–190+) among patients receiving CTLA-4 inhibitor therapy [5]. The study by Rahimi et al., which examined chemo-immunotherapy following anti-PD-1 failure, reported a median OS of 69.5 weeks (range, 30.4–108.6) [17]. OS was not reported in studies examining RP1 oncolytic virotherapy, TLR9 agonist therapy, dostarlimab, or HER2 antagonist therapy [14,15,18,26].

### 3.4. Progression-Free Survival

Progression-free survival (PFS) was reported in all seven studies examining EGFR-containing therapy. Al-Sharif et al. reported a median PFS of 23 weeks (range, 2.3–64.6+) [5], while Bossi et al. and Becker et al. reported median PFS values of 16 weeks (95% CI, 12.6–22.2) and 39.1 weeks (95% CI, 12.2–45.2), respectively [6,34]. Marin-Acevedo et al. reported a median PFS of 75.2 weeks (95% CI, 19.4–NR) [33]. In the smaller reports, Morecroft et al., Hober et al., and Hsu et al. reported PFS values of 80.4 weeks (range, 46.5–114.3), 121.7 weeks, and 78.2 weeks (range, 26.1–104.2), respectively [35,36,37]. Al-Sharif et al. also reported a median PFS of 23 weeks (range, 4–190+) among patients receiving ipilimumab therapy [5]. The median PFS in Schadendorf et al. was not reported; instead, individual time-on-study data were presented graphically in a swimmer [26]. Milhem et al. reported a median PFS of 12.1 weeks (95% CI 9.3–14.9) in the TLR9 agonist cohort [14]. Among patients who received chemo-immunotherapy, Rahimi et al. reported a median PFS of 28.2 weeks (range, 26.1–30.4) [17]. Gandarillas et al. reported a PFS of 52.1 weeks following dostarlimab therapy [18]. Finally, Strickley et al. reported a PFS of 26.1 weeks with lapatinib-containing therapy [15].

### 3.5. Response Rate (CR/PR, SD and PD)

The overall response rate (ORR) including CR/PR, alongside SD and PD, was reported in all cohorts. The EGFR-based cohort within Al-Sharif et al.’s study was reported to have an ORR of 31% (*n* = 4/13), with one CR (8%) and thee PRs (23%); four patients (31%) reached SD and five (38%) suffered PD [5]. Bossi et al. reported an ORR of 43% (10/23), comprising three CRs (13%) and seven PRs (30%), with SD and PD observed in eight (35%) and five (22%) patients, respectively [6]. Becker et al. reported an ORR of 20% (2/10), with one CR (10%) and one PR (10%); four patients (40%) had SD and four (40%) had PD [34]. Marin-Acevedo et al. reported an ORR of 54% (7/13), comprising one CR (8%) and six PRs (46%), while three patients (23%) each experienced SD and PD [33]. Higher response rates were reported in the smaller series: Morecroft et al. observed an ORR of 100% (3/3), comprising two CRs and one PR, while Hober et al. and Hsu et al. each reported an ORR of 100%, with CRs observed in 1/1 and 3/3 patients, respectively [35,36,37].

The tumour response rate was also reported for the remaining treatment modalities. In the CTLA-4 inhibitor cohort reported by Al-Sharif et al., ORR was 33% (4/12), comprising three CRs (25%) and one PR (8%); one patient (8%) achieved SD and seven (58%) had PD [5]. Schadendorf et al. reported an ORR of 15% (6/39) following RP1 oncolytic virotherapy in combination with nivolumab, comprising four CRs (10%) and two PRs (5%); SD and PD were reported in 14 (36%) and 13 (33%) patients, respectively [26]. No objective responses were observed among the five patients treated with the TLR9 agonist, cavrotolimod alongside cemiplimab, with one patient (20%) achieving SD, three (60%) experiencing PD, and the remaining patient being non-evaluable (20%) [14]. Rahimi et al. reported an ORR of 100% (2/2) following chemo-immunotherapy, with both patients achieving a PR [17]. Complete responses were reported in the single patients treated with alternative anti-PD-1 therapy using dostarlimab [18] and HER2 antagonist therapy using lapatinib in combination with nivolumab [15], corresponding to an ORR of 100% in each case.

### 3.6. Ongoing Trials

A total of eight ongoing clinical trials [25,39,40,42,43,44,47,48] investigating cSCC treatment following anti-PD-1 immunotherapy failure were identified through the ClinicalTrials.gov search engine [23]. At the time of the updated search, four trials were recruiting [40,42,43,47] and four were active but not recruiting [25,39,44,48]. The median confirmed or estimated sample size was 89 participants (range, 23–398). Among the eight included trials, therapeutic approaches were heterogeneous. All eight trials required prior treatment failure or refractoriness, either specifically to anti-PD-1/PD-L1 therapy or to available standard-of-care systemic therapy, which for advanced cSCC, is currently centred on anti-PD-1 immunotherapy. The majority are evaluating immunotherapy-based strategies delivered either orally [43], intravenously [42,44], or intra-tumourally [40,47], with several studies also comparing this to combination approaches involving targeted therapy [42], oncolytic viruses [25,44], or mixed-route immunotherapy [40]. Only one trial investigated brachytherapy alone [39] and another is investigating photoimmunotherapy [48].

A wide variety of agents were identified within the eight ongoing trials. The majority were typical and/or atypical anti-PD-1 immunotherapy agents, specifically cemiplimab [40,48] and nivolumab [44]. One trial is exploring the combination immunotherapy (anti-PD-1/anti-vascular endothelial growth factor (VEGF)) agent, ivonescimab [43]. The agents used for immunocytokine therapy, brachytherapy and photoimmunotherapy were daromun (L19IL2/L19TNF), radium-224 and IR700, respectively [39,47,48]. Oncolytic virotherapy agents included vusolimogene oderparepvec (RP1) and talimogene laherparepvec (T-VEC) [25,44]. Cetuximab was the only EGFR inhibitor therapy identified [42], with it being in its ASP-1929 formulation in one trial [48]. Additionally, one trial is investigating HER-family-targeted therapy with HMBD-001 [42].

## 4. Discussion

This systematic review aimed to explore the currently available literature on the management of advanced cutaneous squamous cell carcinoma following anti-PD-1 immunotherapy failure. While the review is limited by the small number and size of the studies published to date, it provides insights into potential treatment options for this patient population.

Among the published literature identified, EGFR-containing treatment strategies, particularly cetuximab-based regimens, represent the most frequently reported salvage approach following anti-PD-1 failure. Across seven studies comprising 66 patients, objective responses and periods of disease control were reported, suggesting clinically meaningful treatment activity in a proportion of patients with advanced cSCC following anti-PD-1 failure [5,6,33,34,35,36,37]. These findings are biologically plausible given the frequent expression of EGFR in cSCC and the fact that EGFR inhibition acts through a mechanistically distinct pathway from PD-1 blockade. Although the reported PFS values were generally numerically favourable compared with those reported for RP1 oncolytic virotherapy (*n* = 39), CTLA-4 inhibition (*n* = 12), TLR9 agonist therapy (*n* = 5), chemo-immunotherapy (*n* = 2), dostarlimab (*n* = 1) and HER-2 antagonist therapy (*n* = 1), these comparisons are indirect, non-randomised and involve markedly unequal treatment cohorts. It therefore cannot establish comparative superiority of EGFR-based therapy over alternative approaches.

In addition, the EGFR inhibitor cohort was highly heterogeneous; only 14 patients (21%) received EGFR inhibitor monotherapy, while the remaining 52 patients received combination regimens. Thirty-nine patients (59%) received an EGFR inhibitor with concurrent anti-PD-1 immunotherapy, including pembrolizumab-containing regimens in 28 patients (42%), avelumab in 10 (15%), and nivolumab in 1 (1.5%). The remaining combination regimens comprised cetuximab with carboplatin and paclitaxel (*n* = 6; 9%), radiotherapy (*n* = 6; 9%), or carboplatin alone (*n* = 1; 1.5%). Clinical outcomes for patients receiving EGFR monotherapy were not reported separately, precluding direct comparison with combination regimens. Consequently, the apparent clinical benefit observed in PFS should be interpreted as reflecting EGFR-based treatment strategies rather than EGFR inhibitor monotherapy alone. This highlights an important gap in the current evidence base, as it remains unclear whether EGFR inhibition is most effective when delivered as monotherapy, in combination with continued PD-1 blockade, or alongside chemoradiotherapy following anti-PD-1 failure. Notably, two of the eight ongoing studies include cetuximab and may provide important prospective data.

Beyond EGFR-containing treatment, a number of alternative strategies have been utilised in the literature, although evidence remains limited and is largely derived from small cohorts and/or case reports. Schadendorf et al. reported outcomes for 39 patients with anti-PD-1-failed cSCC treated with RP1 oncolytic virotherapy in combination with nivolumab. An objective response was observed in six patients (15%), including four CRs (10%) and two PRs (5%), while 14 patients (36%) achieved SD [26]. These findings suggest that RP1-based therapy may have activity in a subset of patients with anti-PD-1-refractory cSCC, although interpretation is limited by the absence of a comparator group, concurrent nivolumab therapy, and the fact that the findings have currently only been reported in conference-poster form.

Evidence for other reported treatment modalities was substantially more limited. In Al-Sharif et al., ipilimumab-containing therapy was administered to 12 patients, of whom 4 (33.3%) achieved an objective response, including 3 CRs and 1 PR; however, the small cohort and inclusion of one patient receiving concurrent nivolumab substantially limit the generalisability of these findings [5]. Milhem et al. reported no objective responses among five patients treated with the TLR9 agonist cavrotolimod in combination with cemiplimab, with one patient achieving SD and three experiencing PD, while one patient was non-evaluable [14]. Three additional treatment approaches were represented only by small case reports. Rahimi et al. reported PRs in two patients treated with carboplatin and paclitaxel alongside continued immunotherapy, while Gandarillas et al. described a CR to dostarlimab following prior cemiplimab failure, and Strickley et al. reported a CR to lapatinib combined with nivolumab [15,17,18]. Although these observations broaden the range of potential salvage strategies described in the literature, their very small sample sizes and frequent use of combination therapy mean that they should be regarded as hypothesis-generating rather than evidence of treatment efficacy or superiority.

The direction of current research is heavily focused on immunotherapy-based treatments, centred on immunotherapy rechallenge, combination immunotherapy and immune-modulating strategies. One possible explanation for this emphasis is that anti-PD-1 immunotherapy has demonstrated more frequent and more durable tumour control than cetuximab-based therapy, which likely contributed to immune checkpoint inhibition replacing cetuximab as first-line treatment for unresectable advanced cSCC. However, optimal management after anti-PD-1 failure remains undefined for the substantial proportion of patients who are unresponsive to treatment. Future studies should therefore investigate whether prior exposure to anti-PD-1 therapy influences the efficacy of subsequent EGFR inhibition. It is also encouraging that a number of mechanistically distinct approaches, including oncolytic virotherapy, photoimmunotherapy and anti-human epidermal growth factor receptor 3 (HER3) therapies, are also being explored, as these may offer additional life-prolonging treatment for patients with anti-PD-1-refractory disease.

This review has several strengths. The protocol was registered on PROSPERO, supporting methodological transparency. A comprehensive search strategy was applied across PubMed, MEDLINE, and Scopus, improving the reliability and reproducibility of the findings. Internal validity was strengthened by the use of predefined inclusion and exclusion criteria, independent screening by two authors, and the use of multiple JBI critical appraisal tools to identify and exclude studies with substantial methodological concerns. In addition, the review incorporated both published clinical evidence and ongoing clinical trials, allowing comparison between the current evidence base and the prospective direction of research.

Despite several important observations, this review is subject to a number of limitations. Foremost, the overall evidence base remains small, with very limited patient numbers within several treatment cohorts and a predominance of non-randomised study designs, restricting both internal validity and generalisability. Treatment heterogeneity is a further important limitation, as many patients received combination regimens rather than monotherapy, meaning that observed effects on OS, PFS and tumour response cannot be attributed to an individual treatment modality. Although this reflects real-world clinical practice, it complicates interpretation and limits the ability to determine the independent contribution of specific agents. In addition, within Al-Sharif et al., four patients contributed to more than one treatment category, further precluding valid direct comparisons between modalities [5].

There was also considerable heterogeneity in response definitions, assessment methods, and assessment time points across the included studies, while incomplete and non-separable outcome reporting reduced the consistency and completeness of the available evidence. These factors, together with the small and unequal treatment cohorts, overlapping participants, and heterogeneous study designs, meant that valid comparative efficacy testing between treatment strategies could not be performed. Similarly, the available survival data could not be appropriately pooled, as study-specific median PFS and OS estimates were derived from heterogeneous populations with differing follow-up durations and censoring patterns; consequently, survival outcomes were reported at the study level rather than as pooled estimates. Some of the evidence, particularly for RP1 oncolytic virotherapy, was available only in conference or poster form and had therefore not yet undergone full peer-reviewed publication, which further limits confidence in the completeness of the reported findings. The apparent prominence of EGFR-containing treatment strategies in the current literature may also reflect publication and data-availability bias rather than true comparative superiority, particularly given that several alternative strategies are represented only by small cohorts or case reports, while many ongoing immunotherapy-focused trials have yet to report clinical outcomes. Furthermore, although database searches were not restricted by language, only English-language reports were eligible for inclusion, potentially excluding relevant evidence.

## 5. Conclusions

While limited by the small numbers of studies and patients reported to date, this review indicates that EGFR-containing strategies, particularly cetuximab-based regimens, are the most frequently reported salvage approach following anti-PD-1 failure and demonstrate preliminary evidence of treatment activity in advanced cSCC. However, the available evidence is derived from small, heterogeneous, non-randomised cohorts in which combination regimens are frequently used and is insufficient to establish superiority over alternative treatment strategies. Evidence for CTLA-4 inhibition, TLR9 agonism, oncolytic virotherapy, chemo-immunotherapy, dostarlimab and HER2 antagonism remains limited by small cohorts. Ongoing clinical trials are predominantly focused on immunotherapy-based approaches, including combinations of immune checkpoint inhibitors, alongside oncolytic virotherapy, small-molecule inhibitors, immunocytokine therapy, and other novel strategies. These studies will be important in defining the optimal management of patients with advanced cSCC following anti-PD-1 treatment failure.

## Data Availability

No new data were created or analysed in this study. Data sharing is not applicable to this article.

## Supplementary Materials

The following supporting information can be downloaded at https://www.mdpi.com/article/10.3390/cancers18183060/s1, File S1: PRISMA 2020 checklist; Table S1: PubMed search strategy; Table S2: MEDLINE search strategy; Table S3: Scopus search strategy; Table S4: Risk of bias assessment of prospective non-randomised single-arm interventional studies using the Joanna Briggs Institute critical appraisal checklist for quasi-experimental studies tool summary; Table S5: Risk of bias assessment of cohort studies using the Joanna Briggs Institute checklist for cohort studies summary; Table S6: Risk of bias assessment of case reports using the Joanna Briggs Institute checklist for case reports summary; Table S7: Study characteristics; Table S8: Outcomes Of post-anti-PD-1 failure treatment; Table S9: Ongoing clinical trials investigating management options following anti-PD-1 failure.

## References

[1] Sun H., Fu J., Li J., Yuan W., Xin J. (2026). Global, regional and national burden of skin cancer from 1990 to 2021: Analysis of risk factors and prediction of trends in 2035. Medicine.

[2] Jiang R., Fritz M., Que S.K.T. (2024). Cutaneous Squamous Cell Carcinoma: An Updated Review. Cancers.

[3] National Institute for Health and Care Excellence (NICE) (2025). Skin Cancers—Recognition and Referral: How Common Is It? NICE Clinical Knowledge Summaries: NICE. https://cks.nice.org.uk/topics/skin-cancers-recognition-referral/background-information/prevalence/.

[4] Huynh J., Eigentler T., Moritz R.K.C., Poch G., Schlaak M., Dobos G. (2025). Cetuximab plus 5-fluorouracil in patients with advanced cutaneous squamous cell carcinoma: A retrospective cohort study. J. Dtsch. Dermatol. Ges..

[5] AlSharif H., Danchine V., Deekollu K., Hasanov M., Wu R., Haykal T., Faieta A., Verschraegen C.F. (2026). Second-line treatments for patients with programmed cell death protein 1-refractory cutaneous squamous cell carcinomas: A brief report. Oncologist.

[6] Bossi P., Alberti A., Bergamini C., Resteghini C., Locati L.D., Alfieri S., Cavalieri S., Colombo E., Gurizzan C., Lorini L. (2025). Immunotherapy followed by cetuximab in locally advanced/metastatic cutaneous squamous cell carcinomas: The I-TACKLE trial. Eur. J. Cancer..

[7] Lin X., Kang K., Chen P., Zeng Z., Li G., Xiong W., Yi M., Xiang B. (2024). Regulatory mechanisms of PD-1/PD-L1 in cancers. Mol. Cancer.

[8] Hughes B.G.M., Guminski A., Bowyer S., Migden M.R., Schmults C.D., Khushalani N.I., Chang A.L.S., Grob J.J., Lewis K.D., Ansstas G. (2025). A phase 2 open-label study of cemiplimab in patients with advanced cutaneous squamous cell carcinoma (EMPOWER-CSCC-1): Final long-term analysis of groups 1, 2, and 3, and primary analysis of fixed-dose treatment group 6. J. Am. Acad. Dermatol..

[9] Dong Q., Zhang Z., Li S., Liang L. (2025). Mechanisms of immunotherapy in cutaneous squamous cell carcinoma in the tumor microenvironment. Front. Immunol..

[10] Baruch E.N., Gleber-Netto F.O., Nagarajan P., Rao X., Akhter S., Eichwald T., Xie T., Balood M., Adewale A., Naara S. (2025). Cancer-induced nerve injury promotes resistance to anti-PD-1 therapy. Nature.

[11] Singh H., Chopra H., Singh I., Mohanto S., Ahmed M.G., Ghumra S., Seelan A., Survase M., Kumar A., Mishra A. (2024). Molecular targeted therapies for cutaneous squamous cell carcinoma: Recent developments and clinical implications. EXCLI J..

[12] Hossen M.M., Ma Y., Yin Z., Xia Y., Du J., Huang J.Y., Huang J.J., Zou L., Ye Z., Huang Z. (2023). Current understanding of CTLA-4: From mechanism to autoimmune diseases. Front. Immunol..

[13] Ward F.J., Kennedy P.T., Al-Fatyan F., Dahal L.N., Abu-Eid R. (2025). CTLA-4-two pathways to anti-tumour immunity?. Immunother. Adv..

[14] Milhem M.M., Wise-Draper T.M., Chandra S., Hanna G.J., Laux D.E., Medina T.M., Ansstas G., Daud A., Kelly C.M., O’Day S.J. (2025). Phase 1b/2 study evaluating safety, efficacy and immune effects of TLR9 agonist cavrotolimod with anti-PD-1 antibodies among patients with advanced solid tumors. J. Immunother. Cancer.

[15] Strickley J.D., Spalding A.C., Haeberle M.T., Brown T., Stevens D.A., Jung J. (2018). Metastatic squamous cell carcinoma of the skin with clinical response to lapatinib. Exp. Hematol. Oncol..

[16] Thomas S., Kuncheria L., Roulstone V., Kyula J.N., Mansfield D., Bommareddy P.K., Smith H., Kaufman H.L., Harrington K.J., Coffin R.S. (2019). Development of a new fusion-enhanced oncolytic immunotherapy platform based on herpes simplex virus type 1. J. Immunother. Cancer.

[17] Rahimi G., In G.K. (2025). Chemoimmunotherapy in Advanced, PD-1 Refractory Cutaneous Squamous Cell Carcinoma: Insights From Two Immunocompromised Patient Cases. Case Rep. Oncol. Med..

[18] Gandarillas S., Tang H., Dasgeb B. (2024). Case Report: Dostarlimab for treatment of aggressive cutaneous squamous cell carcinoma. Front. Med..

[19] Page M.J., McKenzie J.E., Bossuyt P.M., Boutron I., Hoffmann T.C., Mulrow C.D., Shamseer L., Tetzlaff J.M., Akl E.A., Brennan S.E. (2021). The PRISMA 2020 statement: An updated guideline for reporting systematic reviews. BMJ.

[20] Riva J., Malik K., Burnie S., Endicott A., Busse J. (2012). What is your research question? An introduction to the PICOT format for clinicians. J. Can. Chiropr. Assoc..

[21] Dissemination CfRa PROSPERO: International Prospective Register of Systematic Reviews: National Institute for Health and Care Research (NIHR). https://www.crd.york.ac.uk/PROSPERO/home.

[22] Thomson H., Sacco J. (2026). Optimal Management of Advanced Cutaneous Squamous Cell Carcinoma Following Anti-PD-1 Immunotherapy Failure PROSPERO: PROSPERO. https://www.crd.york.ac.uk/PROSPERO/view/CRD420261340230.

[23] (US) NLoM (2026). ClinicalTrials.gov: U.S. National Library of Medicine. https://clinicaltrials.gov.

[24] Ouzzani M., Hammady H., Fedorowicz Z., Elmagarmid A. (2016). Rayyan—A web and mobile app for systematic reviews: Qatar Computing Research Institute (QCRI). Syst. Rev..

[25] Replimune I. (2018). An Open-Label, Multicenter, Phase 1/2 Study of RP1 as a Single Agent and in Combination with PD1 Blockade in Patients with Solid Tumors [IGNYTE]: ClinicalTrials.gov. Updated 19 March 2026. https://clinicaltrials.gov/study/NCT03767348.

[26] Schadendorf D., Wong M., Beasley G., Pavlick A., Harrington K., Chmielowski B., Niu J., VanderWalde A., Bowles T., Tsai K. Efficacy and safety of RP1 plus nivolumab in patients with non-melanoma skin cancer. Proceedings of the 22nd European Association of Dermato-Oncology (EADO) Congress.

[27] Xue L., Han Y., Zhang Q., Li X., Fang M., Zhong L., Wang S., Liu Y., Zhang S., Li Z. (2026). Becotatug Vedotin in Patients with Recurrent or Metastatic Squamous Cell Carcinoma of Head and Neck: A Multicenter, Phase IIa Trial. Clin. Cancer Res..

[28] Sato M., Enokida T., Wada A., Okano S., Tanaka H., Fujisawa T., Ueda Y., Motegi A., Shinozaki T., Takeshita N. (2023). Potential efficacy of local therapy for progressive lesions after nivolumab in patients with recurrent or metastatic squamous cell carcinoma of the head and neck. Int. J. Clin. Oncol..

[29] Kassardjian A.A., Ladbury C.J., Tam A., Maroongroge S., Xing Y., Muddasani R., Modi B., Amini A. (2025). Outcomes and toxicity of concomitant radioimmunotherapy following PD-1 blockade for locally advanced and metastatic cutaneous squamous cell carcinoma. Front. Oncol..

[30] Saleh K., Daste A., Martin N., Pons-Tostivint E., Auperin A., Herrera-Gomez R.G., Baste-Rotllan N., Bidault F., Guigay J., Le Tourneau C. (2019). Response to salvage chemotherapy after progression on immune checkpoint inhibitors in patients with recurrent and/or metastatic squamous cell carcinoma of the head and neck. Eur. J. Cancer.

[31] Kurosaki T., Mitani S., Tanaka K., Suzuki S., Kanemura H., Haratani K., Fumita S., Iwasa T., Hayashi H., Yoshida T. (2021). Safety and efficacy of cetuximab-containing chemotherapy after immune checkpoint inhibitors for patients with squamous cell carcinoma of the head and neck: A single-center retrospective study. Anticancer Drugs.

[32] Tanaka H., Enokida T., Okano S., Fujisawa T., Tanaka N., Takeshita N., Onaga R., Hoshi Y., Wada A., Sato M. (2023). Subsequent chemotherapy with paclitaxel plus cetuximab-based chemotherapy following immune checkpoint inhibitor in recurrent or metastatic squamous cell carcinoma of the head and neck. Front. Oncol..

[33] Marin-Acevedo J.A., Withycombe B.M., Kim Y., Brohl A.S., Eroglu Z., Markowitz J., Tarhini A.A., Tsai K.Y., Khushalani N.I. (2023). Cetuximab for Immunotherapy-Refractory/Ineligible Cutaneous Squamous Cell Carcinoma. Cancers.

[34] Becker J.C., Gesierich A.H., Leiter U., Zimmer L., Hassel J.C., von Wasielewski I., Ziemer M., Fluck M., Meier F., Spillner A.N. (2025). Avelumab plus cetuximab in patients with unresectable stage III or IV cutaneous squamous cell carcinoma: Clinical activity and safety results from AliCe, a single-arm multicentre phase II DeCOG trial. Br. J. Dermatol..

[35] Morecroft R., Phillipps J., Zhou A., Butt O., Khaddour K., Johanns T., Ansstas G. (2024). Case report: Increased efficacy of cetuximab after pembrolizumab failure in cutaneous squamous cell carcinoma. Front. Oncol..

[36] Hsu E., Eton O., Patel A.V., Cartun R., Earle J.S., Mnayer L.O., Kotowitz J., Yu P.P. (2021). Combining Panitumumab with Anti-PD-1 Antibody in Cutaneous Squamous Cell Carcinoma of the Head and Neck After Inadequate Response to Anti-PD-1 Antibody Alone. J. Drugs Dermatol..

[37] Hober C., Jamme P., Desmedt E., Greliak A., Mortier L. (2021). Dramatic response of refractory metastatic squamous cell carcinoma of the skin with cetuximab/pembrolizumab. Ther. Adv. Med. Oncol..

[38] AbbVie (2016). A Multicenter, Phase 1, Open-Label, Dose-Escalation Study of ABBV-927 and ABBV-181, an Immunotherapy, in Subjects with Advanced Solid Tumors: ClinicalTrials.gov. https://clinicaltrials.gov/study/NCT02988960.

[39] Alpha Tau Medical (2022). A Prospective International Multicenter, Pivotal, Single Arm, Open Label Clinical Study to Assess the Efficacy and Safety of Intratumoral Alpha DaRT224 for the Treatment of Patients with Recurrent Cutaneous Squamous Cell Carcinoma: ClinicalTrials.gov. https://clinicaltrials.gov/study/NCT05323253.

[40] Ankyra Therapeutics (2023). A Phase I Open-Label, Dose Escalation Study of the Safety and Tolerability of Tolododekin Alfa (ANK-101) in Advanced Solid Tumors: ClinicalTrials.gov. https://clinicaltrials.gov/study/NCT06171750.

[41] Emory University (2025). Phase 1b Study of TMV Vaccine Therapy Alone and TMV Vaccine Plus Pembrolizumab for Recurrent and/or Metastatic Head and Neck Squamous Cell Carcinoma (HNSCC): ClinicalTrials.gov. https://clinicaltrials.gov/study/NCT06868433.

[42] Hummingbird Bioscience (2023). Study of an Anti-HER3 Antibody, HMBD-001, with Docetaxel +/- Cetuximab in Advanced Squamous Non-small Cell Lung Cancers, and HMBD-001 + Cetuximab in Advanced Squamous Cell Cancers: ClinicalTrials.gov. https://clinicaltrials.gov/study/NCT05910827.

[43] M.D. Anderson Cancer Center. (2024). Phase 2 Study of Ivonescimab in Patients with Cutaneous Squamous Cell Carcinoma: ClinicalTrials.gov. https://clinicaltrials.gov/study/NCT06567314.

[44] National Cancer Institute (2016). A Phase II Study of Talimogene Laherparepvec Followed by Talimogene Laherparepvec + Nivolumab in Refractory T Cell and NK Cell Lymphomas, Cutaneous Squamous Cell Carcinoma, Merkel Cell Carcinoma, and Other Rare Skin Tumors: ClinicalTrials.gov. https://clinicaltrials.gov/study/NCT02978625.

[45] National Cancer Institute (2021). A Phase II/III Trial of Chemotherapy + Cetuximab vs Chemotherapy + Bevacizumab vs Atezolizumab + Bevacizumab Following Progression on Immune Checkpoint Inhibition in Recurrent/Metastatic Head and Neck Cancers. https://clinicaltrials.gov/study/NCT05063552.

[46] National Cancer Institute (2020). Phase I Trial of BAY 1895344 ATR Inhibitor Combined with Stereotactic Body Radiation Therapy and Pembrolizumab for Recurrent Head and Neck Squamous Cell Carcinoma: ClinicalTrials.gov. https://clinicaltrials.gov/study/NCT04576091.

[47] Philogen S.p.A. (2025). A Phase 2 Study of Intratumoral Administration of L19IL2/L19TNF in Locally Advanced Cutaneous Squamous Cell Carcinoma Patients Progressing on or Intolerant to Systemic Treatment: ClinicalTrials.gov. https://clinicaltrials.gov/study/NCT07228442.

[48] Rakuten Medical (2020). An Open-Label Study Using ASP-1929 Photoimmunotherapy in Combination with Anti-PD1 Therapy in EGFR Expressing Advanced Solid Tumors: ClinicalTrials.gov ID. https://clinicaltrials.gov/study/NCT04305795.

[49] Barker T.H., Habibi N., Aromataris E., Stone J.C., Leonardi-Bee J., Sears K., Hasanoff S., Klugar M., Tufanaru C., Moola S. (2024). The revised JBI critical appraisal tool for the assessment of risk of bias for quasi-experimental studies. JBI Evid. Synth..

[50] Barker T.H., Hasanoff S., Aromataris E., Stone J.C., Leonardi-Bee J., Sears K., Habibi N., Klugar M., Tufanaru C., Moola S. (2025). The revised JBI critical appraisal tool for the assessment of risk of bias for cohort studies. JBI Evid. Synth..

[51] Moola S., Munn Z., Sears K., Sfetcu R., Currie M., Lisy K., Tufanaru C., Qureshi R., Mattis P., Mu P. (2015). Conducting systematic reviews of association (etiology): The Joanna Briggs Institute’s approach. Int. J. Evid. Based Healthc..

[52] (2026). Microsoft Excel.

