# Supplementary material for: Treatment Strategies for Advanced Cutaneous Squamous Cell Carcinoma Following Anti-PD-1 Immunotherapy Failure: A Systematic Review of the Literature and Ongoing Trials
Source: Cancers (Basel). 2026 Sep 21;18(18):3060. doi: 10.3390/cancers18183060 (PMC13605999; doi:10.3390/cancers18183060)
Supplement: Supplementary file 1 [file cancers-18-03060-s001.zip › cancers-4513446-supplementary.pdf]

Supplementary Tables

Table S1. PubMed search strategy.

Date – 27/8/2026

Number	Term	Results
#1	(cutaneous squamous cell carcinoma*[tiab] OR cSCC[tiab] OR CSCC[tiab] OR squamous cell carcinoma of the skin[tiab] OR skin squamous cell carcinoma[tiab] OR cutaneous SCC[tiab] OR metastatic cutaneous squamous cell carcinoma[tiab] OR metastatic squamous cell carcinoma[tiab] OR advanced cutaneous squamous cell carcinoma[tiab] OR unresectable cutaneous squamous cell carcinoma[tiab] OR locally advanced cutaneous squamous cell carcinoma[tiab])	8,496
#2	(“Programmed Cell Death 1 Receptor”[Mesh] OR “Programmed Cell Death 1 Ligand 1 Protein”[Mesh] OR PD-1[tiab] OR anti-PD-1[tiab] OR PD1[tiab] OR programmed cell death 1[tiab] OR programmed death 1[tiab] OR PD-L1[tiab] OR anti-PD-L1[tiab] OR immune checkpoint inhibitor*[tiab] OR checkpoint inhibitor*[tiab] OR immunotherapy[tiab] OR cemiplimab[tiab] OR pembrolizumab[tiab] OR nivolumab[tiab] OR avelumab[tiab])	258,203
#3	(fail*[tiab] OR failure[tiab] OR refractory[tiab] OR resist*[tiab] OR progressive[tiab] OR progression[tiab] OR post-progression[tiab] OR salvage[tiab] OR second-line[tiab] OR subsequent-line[tiab] OR inadequate response[tiab] OR unresponsive[tiab] OR after immunotherapy[tiab] OR following immunotherapy[tiab] OR after anti-PD-1[tiab] OR following anti-PD-1[tiab] OR treatment resistant[tiab] OR after[tiab] OR following[tiab] OR unresectable[tiab])	10,132,578
#4	(management[tiab] OR treatment[tiab] OR therapy[tiab] OR therapeutic[tiab] OR salvage therapy[tiab] OR systemic therapy[tiab] OR chemotherapy[tiab] OR chemoimmunotherapy[tiab] OR cetuximab[tiab] OR panitumumab[tiab] OR 5-fluorouracil[tiab] OR fluorouracil[tiab] OR EGFR inhibitor[tiab] OR anti-EGFR[tiab] OR radiotherapy[tiab] OR surgery[tiab] OR resection[tiab])	10,882,074
#5	#1 AND #2 AND #3 AND #4	628
#6	#1 AND #2 AND #3 AND #4 Filters: from 2000/1/1 – 2026/08/27	628

Table S2. MEDLINE search strategy.

Date – 27/8/2026

Number	Term	Results
#1	(cutaneous squamous cell carcinoma* OR cSCC OR CSCC OR squamous cell carcinoma of the skin OR skin squamous cell carcinoma OR cutaneous SCC OR metastatic cutaneous squamous cell carcinoma OR metastatic squamous cell carcinoma OR advanced cutaneous squamous cell carcinoma OR unresectable cutaneous squamous cell carcinoma OR locally advanced cutaneous squamous cell carcinoma).ti,ab.	8,510
#2	exp Programmed Cell Death 1 Receptor/ OR exp Programmed Cell Death 1 Ligand 1 Protein/ OR (PD-1 OR anti-PD-1 OR PD1 OR programmed cell death 1 OR programmed death 1 OR PD-L1 OR anti-PD-L1 OR immune checkpoint inhibitor* OR checkpoint inhibitor* OR immunotherapy OR cemiplimab OR pembrolizumab OR nivolumab OR avelumab).ti,ab.	239,194
#3	(fail* OR failure OR refractory OR resist* OR progressive OR progression OR post-progression OR salvage OR second-line OR subsequent-line OR inadequate response OR unresponsive OR after immunotherapy OR following immunotherapy OR after anti-PD-1 OR following anti-PD-1 OR treatment resistant OR after OR following OR unresectable).ti,ab.	11,092,121
#4	(management OR treatment OR therapy OR therapeutic OR salvage therapy OR systemic therapy OR chemotherapy OR chemoimmunotherapy OR cetuximab OR panitumumab OR 5-fluorouracil OR fluorouracil OR EGFR inhibitor OR anti-EGFR OR radiotherapy OR surgery OR resection).ti,ab.	10,617,273
#5	#1 AND #2 AND #3 AND #4	578
#6	limit 5 to yr=”2000 -Current”	578

Table S3. Scopus search strategy.

Date – 27/8/2026

Number	Term	Results
**#1**	TITLE-ABS-KEY("cutaneous squamous cell carcinoma*" OR "cSCC" OR "CSCC" OR "squamous cell carcinoma of the skin" OR "skin squamous cell carcinoma" OR "cutaneous SCC" OR "metastatic cutaneous squamous cell carcinoma" OR "advanced cutaneous squamous cell carcinoma" OR "unresectable cutaneous squamous cell carcinoma" OR "locally advanced cutaneous squamous cell carcinoma")	8,398
**#2**	TITLE-ABS-KEY("PD-1" OR "anti-PD-1" OR "PD1" OR "programmed cell death 1" OR "programmed death 1" OR "PD-L1" OR "anti-PD-L1" OR "immune checkpoint inhibitor*" OR "checkpoint inhibitor*" OR "cemiplimab" OR "pembrolizumab" OR "nivolumab" OR "avelumab")	177,335
**#3**	TITLE-ABS-KEY("fail" OR "failure" OR "refractory" OR "resist" OR "progression" OR "progressive" OR "salvage" OR "second line" OR "second-line" OR "subsequent line" OR "inadequate response" OR "unresponsive" OR "post progression" OR "after anti-PD-1" OR "following anti-PD-1" OR "after cemiplimab" OR "after" OR "following" OR "unresectable")	19,001,276
**#4**	TITLE-ABS-KEY("management" OR "treatment" OR "therapy" OR "therapeutic" OR "salvage therapy" OR "systemic therapy" OR "chemotherapy" OR "chemoimmunotherapy" OR "cetuximab" OR "panitumumab" OR "5-fluorouracil" OR "fluorouracil" OR "EGFR inhibitor" OR "anti-EGFR" OR "radiotherapy" OR "surgery" OR "resection")	22,494,218
**#5**	#1 AND #2 AND #3 AND #4	518
**#6**	#1 AND #2 AND #3 AND #4 AND PUBYEAR>1999	518

Table S4. Risk-of-bias assessment of prospective non-randomised single-arm interventional studies using the Joanna Briggs Institute critical appraisal checklist for quasi-experimental studies tool summary

Study	Is it clear what is the "cause" and what is the "effect"?	Was there a control group?	Were participants included in any comparisons similar?	Were participants included in any comparisons receiving similar treatment/care other than the intervention of interest?	Were there multiple measurements of the outcome both pre- and post-intervention?	Were the outcomes of participants included in any comparisons measured in the same way?	Were outcomes measured in a reliable way?	Was follow-up complete and if not, were differences between groups in terms of their follow-up adequately described and analysed?	Was appropriate statistical analysis used?	Overall Appraisal
**(Schadendorf, 2026)**	Yes	No	Yes	Yes	Yes	Yes	Yes	Unclear	No	**Include**
**(Bossi, 2025)**	Yes	No	No	Yes	Yes	Yes	Yes	Yes	Yes	**Include**
**(Milhem, 2025)**	Yes	No	Not applicable	Not applicable	Yes	Not applicable	Yes	No	Yes	**Include**
**(Becker, 2025)**	Yes	No	Not applicable	Not applicable	Yes	Yes	Yes	Yes	Yes	**Include**

- Green - Yes
- Red - No
- Yellow - Unclear
- Purple - Not applicable

Table S5. Risk-of-bias assessment of cohort studies using the Joanna Briggs Institute checklist for cohort studies summary

Study	Were the two groups similar and recruited from the same population?	Were the exposures measured similarly to assign people to both exposed and unexposed groups?	Was the exposure measured in a valid and reliable way?	Were confounding factors identified?	Were strategies to deal with confounding factors stated?	Were the groups/participants free of the outcome at the start of the study (or at the moment of exposure)?	Were the outcomes measured in a valid and reliable way?	Was the follow up time reported and sufficient to be long enough for outcomes to occur?	Was follow up complete, and if not, were the reasons to loss to follow up described and explored?	Were strategies to address incomplete follow up utilized?	Was appropriate statistical analysis used?	Overall Appraisal
**(Al-Sharif, 2026)**												**Include**
**(Huynh, 2025)**												**Exclude**
**(Marin-Acevedo, 2023)**												**Include**

- Yes
- No
- Unclear
- Not applicable

Table S6. Risk-of-bias assessment of case reports using the Joanna Briggs Institute checklist for case reports summary

Study	Were patient's demographic characteristics clearly described?	Was the patient's history clearly described and presented as a timeline?	Was the current clinical condition of the patient on presentation clearly described?	Were diagnostic tests or assessment methods and the results clearly described?	Was the intervention(s) or treatment procedure(s) clearly described?	Was the post-intervention clinical condition clearly described?	Were adverse events (harms) or unanticipated events identified and described?	Does the case report provide takeaway lessons?	Overall Appraisal
(Rahimi, 2025)									Include
(Gandarillas, 2024)									Include
(Morecroft, 2024)									Include
(Hober, 2021)									Include
(Hsu, 2021)									Include
(Strickley, 2018)									Include

- Yes
- No
- Unclear

Table S7. Study characteristics

- Study	Location	Single or Multi Centre	Study Type	Sample	Age, Median (Range) or Mean (Range), years	Gender, n (%)	Oncological management following Anti-PD-1 failure, n (%)	Follow-Up, Median (Range), months	Response Assessment (Criteria; Assessment time; evaluable denominator)
(Schadendorf, 2026) [26]	Germany	Multi	Prospective non-randomised single-arm interventional study (Conference poster)	39	69 (38-93)	M – 30 (77)<br>F – 9 (23)	RP1 + Anti-PD-1-I – 39 (100)	NR (0.5-46.3)	Investigator-assessed mRECIST v1.1; assessment schedule NR; n=33 response-evaluable/39 full analysis set (6 NE); ORR denominator n=39
(AlSharif, 2026) [5]	USA	Single	Retrospective analysis	21	Anti-CTLA4-I group – 77 (48-85)<br>EGFR-I group – 73 (42-86)	NR*	Anti-CTLA4-I – 11 (52)<br>Anti-CTLA4-I + Anti-PD-1-I – 1 (8)<br>EGFR-I – 7 (33)**<br>EGFR + CP + PTAX – 6 (29)	NR (12-72)	Clinical judgement (RECIST not used); assessment time NR; n=13 evaluable (EGFR cohort), n=12 evaluable (CTLA-4 cohort)
(Bossi, 2025) [6]	Italy	Multi	Prospective non-randomised single-arm interventional study (Phase II)	23	NR (NR)	NR (NR)	EGFR-I + Anti-PD-1-I – 23 (100)	9 (1-26)	RECIST v1.1; first assessment 6 weeks after cetuximab addition, with repeat assessment within 4 weeks if no PR/CR; n=23 evaluable
(Milhem, 2025) [14]	USA	Multi	Prospective non-randomised single-arm interventional study (Phase Ib/II)	5	Mean – 61.2 (54-67)	M – 3 (60)<br>F – 2 (40)	TLR9A + Anti-PD-1-I – 5 (100)	***	RECIST v1.1; tumour assessment every 12 weeks; n=4 cSCC patients evaluable
(Becker, 2025) [34]	Germany	Multi	Prospective non-randomised single-arm interventional study (Phase II)	10	NR (NR)	NR (NR)	EGFR-I + Anti-PD-1-I – 10 (100)	35 (NR)	RECIST v1.1; first planned efficacy assessment at week 12; n=10 anti-PD-1-refractory patients evaluable
(Rahimi, 2025) [17]	USA	Single	Case report	2	45 (36-54)	M – 2 (100)	CIO – 2 (100)	16 (7-25)	Clinical and radiological assessment; no formal response criteria reported; response documented at ~2–3 months; n=2 evaluable

(Morecroft, 2024) [35]	USA	Single	Case report	3	61 (36-67)	M – 3 (100)	EGFR-I – 1 (33)<br>EGFR-I + CP – 2 (67)	31.3 (13.3-46.1)	Clinical and radiographic assessment; no formal response criteria reported; initial response at 1.9–7.0 months; n=3 evaluable
(Gandarillas, 2024) [18]	USA	Single	Case report	1	80	M – 1 (100)	Alt-Anti-PD-1-I – 1 (100)	12	Clinical/macroscopic assessment; no formal response criteria reported; response after 3 infusions (~9 weeks); n=1 evaluable
(Marin-Acevedo, 2023) [33]	USA	Single	Retrospective analysis	13	72 (54-89)	M – 11 (85)<br>F – 2 (15)	EGFR-I – 7 (54)<br>EGFR-I + RT – 6 (46)	21 (NR)	RECIST v1.1; assessment schedule NR; n=13 post-ICI patients evaluable
(Hober, 2021) [37]	France	Single	Case report	1	64 (NR)	M – 1 (100)	EGFR-I + Anti-PD-1-I – 1 (100)	28 (NR)	Clinical and radiological assessment; no formal response criteria reported; PR at 3 months and PET-defined CR at ~9 months; n=1 evaluable
(Hsu, 2021) [36]	USA	Single	Case report	3	83 (78-90)	M – 1 (33)<br>F – 2 (67)	EGFR-I + Anti-PD-1-I – 3 (100)	18 (15-24)	Clinical, radiological and/or pathological assessment; scans every 3 months; n=3 evaluable
(Strickley, 2018) [15]	USA	Single	Case report	1	80	M – 1 (100)	HER2-A – 1 (100)	6	Clinical and MRI assessment; no formal response criteria reported; clinical response at 2 months and MRI assessment at 6 months; n=1 evaluable

Key – Anti-CTLA4-I – Anti-Cytotoxic T-lymphocyte-associated protein-4 immunotherapy; Anti-PD-1 – Anti-Programmed Cell Death-1; Anti-PD-1-I – Anti-Programmed Cell Death-1 immunotherapy; CIO – Chemo-immunotherapy; CP – Carboplatin; EGFR-I – Epidermal growth factor receptor inhibitor; F – Female; HER2-A – Human epidermal growth factor receptor 2 antagonist; M – Male; Max – Maximum; Min – Minimum; NR – Not reported; RP1 – Vusolimogene oderparepvec; RT – Radiotherapy; ORR – Objective response rate; PTAX – Paclitaxel; TLR9A – Toll-like receptor 9 agonist; USA – United States of America.

* Sex was reported by treatment cohort (cetuximab: M/F 10/3; ipilimumab: M/F 8/4); four patients were included in both cohorts, preventing calculation of a unique-patient sex distribution.

**Four patients were included in both cohorts.

***Median follow-up was not reported. However, individual patient timelines suggest follow-up extending beyond 80 weeks in some cases.

Table S8. Outcomes of post-Anti-PD-1 failure treatment

Study	Sample	Confounding factors, n (%)	Primary site, n (%)	cSCC AJCC stage, n (%)	Prior therapy, n (%)	Best response to prior Anti-PD-1 therapy, n (%)	Post-Anti-PD-1 failure agent (formulation), n (%)	Nature of post-Anti-PD-1 failure agent, n (%)	Response, n (%)	Progression-free survival (PFS), weeks, median (range) or (95% CI)	Overall survival (OS), weeks, median (range) or (95% CI)
**EGFR-I-Containing Therapy**											
(Al-Sharif, 2026) [5]	13*	S – 8 (62)<br>HC – 4 (31)	H&N – 6 (46)<br>NR – 7 (54)	III – 7 (54)<br>IV – 6 (46)	Cemiplimab – 8 (62)<br>US Anti-PD-1-I – 5 (38)<br>Surgery – 8 (62)<br>Radiotherapy – 4 (31)<br>Chemotherapy – 1 (8)<br>Ipilimumab – 2 (15)	PR – 4 (31)<br>SD – 2 (15)<br>PD – 7 (54)	Cetuximab (IVI) – 13 (100)	Monotherapy – 7 (54)<br>+ CP + PTAX – 6 (46)	**ORR – 4 (31)**<br>CR – 1 (8)<br>PR – 3 (23)<br>SD – 4 (31)<br>PD – 5 (38)	23 (2.3-64.6+)	41.7 (12.2-218.6+)
(Bossi, 2025) [6]	23	NR – 23 (100)	NR – 23 (100)	NR – 23 (100)	Pembrolizumab – 23 (100)	PR – 17 (74)<br>SD – 6 (26)	Cetuximab (IVI) – 23 (100)	+ Pembrolizumab – 23 (100)	**ORR – 10 (43)**<br>CR – 3 (13)<br>PR – 7 (30)<br>SD – 8 (35)<br>PD – 5 (22)	16 (95%CI, 12.6-22.2)	NR
(Becker, 2025) [34]	10	NR – 10 (100)	NR – 10 (100)	NR – 10 (100)	Cemiplimab – 7 (70)<br>Pembrolizumab – 2 (20)<br>Cemiplimab + Pembrolizumab – 1 (10)	SD – 4 (40)<br>PD – 6 (60)	Cetuximab (IVI) – 10 (100)	+ Avelumab – 10 (100)	**ORR – 2 (20)**<br>CR – 1 (10)<br>PR – 1 (10)<br>SD – 4 (40)<br>PD – 4 (40)	39.1 (95%CI, 12.2-45.2)	120 (95%CI, 44.8-NR)
(Morecroft, 2024) [35]	3	HC – 1 (33)	H&N – 1 (33)	NR – 3 (100)	Pembrolizumab – 3 (100)	PD – 3 (100)	Cetuximab (IVI) – 3 (100)	+ CP – 1 (33)	**ORR – 3 (100)**	80.4 (46.5-114.3)	NR

			Trunk – 2 (67)		Surgery – 1 (33)<br>Radiotherapy – 1 (33)			+ Pembrolizumab – 1 (33)<br>+ CP + Pembrolizumab – 1 (33)	CR – 2 (67)<br>PR – 1 (33)		
(Marin-Acevedo, 2023) [33]	13	HC – 1 (8)<br>SC – 1 (8)	H&N – 5 (38)<br>Trunk – 2 (15)<br>UE – 3 (23)<br>LE – 3 (23)	III – 3 (23)<br>IV – 10 (77)	US Anti-PD-1-I – 13 (100)<br>Radiotherapy – 6 (46)<br>CP+PTAX – 1 (8)<br>US-CTT – 1 (8)	PD – 13 (100)	Cetuximab (IVI) – 13 (100)	Monotherapy – 7 (54)<br>+ Radiotherapy – 6 (46)	**ORR – 7 (54)**<br>CR – 1 (8)<br>PR – 6 (46)<br>SD – 3 (23)<br>PD – 3 (23)	75.2 (95%CI, 19.4-NR)	NR
(Hober, 2021) [37]	1	NR – 1 (100)	Trunk – 1 (100)	NR – 1 (100)	Pembrolizumab – 1 (100)<br>Surgery – 1 (100)<br>Radiotherapy – 1 (100)<br>Carboplatin – 1 (100)<br>Cetuximab – 1 (100)**	PD – 1 (100)	Cetuximab (IVI) – 1 (100)	+ Pembrolizumab – 1 (100)	**ORR – 1 (100)**<br>CR – 1 (100)	121.7	NR
(Hsu, 2021) [36]	3	NR – 3 (100)	H&N – 3 (100)	NR – 3 (100)	Nivolumab – 1 (33)<br>Pembrolizumab – 2 (67)<br>Surgery – 3 (100)<br>Radiotherapy – 2 (67)	PD – 3 (100)	Panitumumab (IVI) – 3 (100)	+ Nivolumab – 1 (33)<br>+ Pembrolizumab – 2 (67)	**ORR – 3 (100)**<br>CR – 3 (100)	78.2 (26.1-104.2)	NR
**CTLA-4-I-Containing Therapy**											
(Al-Sharif, 2026) [5]	12*	S – 4 (33)<br>HC – 5 (42)	H&N – 7 (58)<br>NR – 5 (42)	III – 3 (25)<br>IV – 9 (75)	Cemiplimab – 5 (42)<br>US Anti-PD-1-I – 7 (58)	PR – 5 (42)<br>SD – 3 (25)<br>PD – 4 (33)	Ipilimumab (IVI) – 12 (100)	Monotherapy – 11 (92)<br>+ Nivolumab – 1 (8)	**ORR – 4 (33)**<br>CR – 3 (25)<br>PR – 1 (8)	23 (4-190+)	45 (7-190+)

					Surgery – 7 (58)<br>Radiotherapy – 6 (50)<br>Chemotherapy – 5 (42)<br>Cetuximab – 2 (17)				SD – 1 (8)<br>PD – 7 (58)		
**Oncolytic Virotherapy**											
(Schadendorf, 2026) [26]	39	NR – 39 (100)	NR – 39 (100)	IV – 29 (74)<br>NR – 10 (26)	US Anti-PD-1-I – 39 (100)	UC – 39 (100)	Vusolimogene oderparepvec (RP1) (ITI) – 39 (100)	+ Nivolumab – 39 (100)	**ORR – 6 (15)**<br>CR – 4 (10)<br>PR – 2 (5)<br>SD – 14 (36)<br>PD – 13 (33)<br>NE – 6 (15)	NR***	NR
**TLR9-A-Containing Therapy**											
(Milhem, 2025) [14]	5	NR – 5 (100)	NR – 5 (100)	III – 2 (40)<br>IV – 3 (60)	US Anti-PD-1-I – 5 (100)	PD – 5 (100)	Cavrotolimod (ITI) – 5 (100)	+ Cemiplimab – 5 (100)	SD – 1 (20)<br>PD – 3 (60)<br>NE – 1 (20)	12.1 (95%CI 9.3-14.9)	NR
**CIO Therapy**											
(Rahimi, 2025) [17]	2	HC – 1 (50)<br>HIV – 1 (50)	H&N – 1 (50)<br>LE – 1 (50)	NR – 2 (100)	Cemiplimab – 2 (100)<br>Surgery – 2 (100)<br>Radiotherapy – 2 (100)<br>Cetuximab – 2 (100)<br>Capecitabine – 2 (100)	PD – 2 (100)	CP+PTAX (IVI) – 2 (100)	+ Pembrolizumab – 1 (50)<br>+ Cemiplimab – 1 (50)	**ORR – 2 (100)**<br>PR – 2 (100)	28.2 (26.1-30.4)	69.5 (30.4-108.6)

					Carboplatin – 1 (50) Talimogene laherparepvec – 1 (50) Imiquimod – 1 (50)						
**Alt-Anti-PD-1 Therapy**											
(Gandarillas, 2024) [18]	1	SC – 1 (100)	H&N – 1 (100)	NR – 1 (100)	Cemiplimab – 1 (100) Pembrolizumab – 1 (100) Surgery – 1 (100) Radiotherapy – 1 (100) Cetuximab – 1 (100)	PD – 1 (100)	Dostarlimab (IVI) – 1 (100)	Monotherapy – 1 (100)	**ORR – 1 (100)** CR – 1 (100)	52.1	NR
**HER-2A Therapy**											
(Strickley, 2018) [15]	1	NR – 1 (100)	H&N – 1 (100)	NR – 1 (100)	Nivolumab – 1 (100) Surgery – 1 (100) Radiotherapy – 1 (100)	PD -1 (100)	Lapatinib (PO) – 1 (100)	+ Nivolumab – 1 (100)	**ORR – 1 (100)** CR – 1 (100)	26.1	NR

Key – AJCC – American Joint Committee on Cancer; Alt-Anti-PD-1-I – Alternative Anti-Programmed Cell Death-1 immunotherapy; Anti-CTLA4-I – Anti-Cytotoxic T-lymphocyte-associated protein-4 immunotherapy; Anti-PD-1 – Anti-Programmed Cell Death-1; Anti-PD-1-I – Anti-Programmed Cell Death-1 immunotherapy; CI – Confidence interval; CIO – Chemo-immunotherapy; CP – Carboplatin; CR – Complete response; cSCC – Cutaneous squamous cell carcinoma; CTT – Clinical trial therapy; EGFR-I – Epidermal growth factor receptor inhibitor; H&N – Head & neck; HC – Haematological compromise; HER2-A – Human epidermal growth factor receptor 2 antagonist; HIV – Human immunodeficiency virus; ITI – Intra-tumoral injection; IVI – Intravenous infusion; LE – Lower extremity; NE – Non-evaluable; NR – Not reported; RP1 – Vusolimogene oderparepvec; ORR – Overall response rate; PD – Progressive disease; PR – Partial response; PTAX – Paclitaxel; RT – Radiotherapy; S – Smoker; SC – Secondary cancer; SD – Stable disease; TLR9A – Toll-like receptor 9 agonist; UC – Unclear; UE – Upper extremity; US – Unspecified.

*Four patients were included in both cohorts.

** Patient was initially treated with carboplatin + cetuximab, then pembrolizumab monotherapy, and then pembrolizumab + cetuximab.

*** Median PFS was not reported; individual time-on-study data were presented graphically in a swimmer plot instead.

Table S9. Ongoing clinical trials investigating management options following Anti-PD-1 failure

NCT Code	Sponsor	Location	Single or Multi Centre	Study Type	Current Sample	cSCC-specific cohort	Anti-PD-1 failure required?	Status	Formulation & Class	Agent (s)	Estimated year of completion
**NCT06567314 [43]**	**M.D. Anderson Cancer Centre**	USA	Single	Phase II Clinical Trial	24 estimated	Cohort 1	Registry permits anti-PD-1-refractory and/or anti-PD-1-naïve patients	Recruiting	Oral immunotherapy	Ivonescimab (AK112) (Anti-PD-1 + Anti-VEGF)	2030
**NCT07228442 [47]**	**Philogen S.p.A.**	USA	Multi	Phase II Clinical Trial	92 estimated	Entire Study	Prior ICI progression required; anti-PD-1 not specified exclusively	Recruiting	ITI immunotherapy	L19IL2/L19TNF (Daromun)	2031
**NCT05323253 [39]**	**Alpha Tau Medical LTD.**	USA, Canada, Israel	Multi	Interventional Study	86 estimated	Entire Study	Failure of ≥1 first-line standard-of-care therapy required, but anti-PD-1 failure not mandated	Active, not recruiting	Brachytherapy	Alpha DaRT224 (Radium-224)	2025
**NCT04305795 [48]**	**Rakuten Medical, Inc.**	USA	Multi	Phase I/II Clinical Trial	23 actual	Sub-study 3	Yes	Active, not recruiting	Photoimmunotherapy	ASP-1929 (Cetuximab (EGFR-I) + IRDye® 700DX (IR700)) + Cemiplimab (Anti-PD-1)	2027
**NCT05910827 [42]**	**Hummingbird Bioscience**	Australia, Moldova, Singapore, South Korea, Taiwan	Multi	Phase I/II Clinical Trial	398 estimated	Arm C	≥1 prior systemic therapy required, but anti-PD-1 failure not	Recruiting	IV immunotherapy + IV EGFR	HMBD-001 (Anti-HER3) + Cetuximab (EGFR-I)	2026

							specifically required				
**NCT02978625 [44]**	**National Cancer Institute**	USA	Multi	Phase II Clinical Trial	68 estimated	Part II SCC-2 expansion cohort	Yes – Progression during or within 6 months of prior PD-1/PD-L1 therapy required	Active, not recruiting	ITI oncolytic virus + IV immunotherapy	Talimogene Laherparepvec + Nivolumab (Anti-PD-1)	2027
**NCT06171750 [40]**	**Ankyra Therapeutics, Inc.**	USA, Canada	Multi	Phase I Clinical Trial	97 estimated	Part 3 cSCC combination cohort	Progression to available standard-of-care therapy required, but prior anti-PD-1 is not specifically mandatory	Recruiting	ITI immunotherapy vs IV combination immunotherapy	Tolododekin alfa (ANK-101) (IL-2-A) vs Tolododekin alfa (ANK-101) (IL-2-A) + Cemiplimab (Anti-PD-1)	2027
**NCT03767348 [25]**	**Replimune, Inc.**	USA, France, Germany, Spain, UK	Multi	Phase II Clinical Trial	340 estimated	Phase 2 anti-PD-1/PD-L1-failed NMSC cohort	Yes – For the relevant anti-PD-1/PD-L1-failed NMSC cohort	Active, not recruiting	ITI oncolytic virus + IV immunotherapy	Vusolimogene Oderparepvec (RP1) + Nivolumab (Anti-PD-1)	2028

Key – Anti-CD40-A – Anti-CD40 agonist; Anti-PD-1 - – Anti-Programmed Cell Death-1; Anti-VEGF – Anti-Vascular Endothelial Growth Factor; Chemo – Chemotherapy; DaRT - Diffusing Alpha-emitters Radiation Therapy; EGFR-I - Epidermal growth factor receptor inhibitor; HER3 - Human Epidermal Growth Factor Receptor 3; ICI – Immune-checkpoint inhibitor; ID – Intradermal; IL-2-A – Interleukin-2-agonist; ITI – Intra-tumoral injection; IV – Intravenous; NMSC – Non-melanoma skin cancer; SRT – Stereotactic radiotherapy; UK – United Kingdom; USA – United States of America
